# ICAM-1 Targeted Drug Combination Nanoparticles Enhanced Gemcitabine-Paclitaxel Exposure and Breast Cancer Suppression in Mouse Models

**DOI:** 10.3390/pharmaceutics14010089

**Published:** 2021-12-31

**Authors:** Linxi Zhu, Qingxin Mu, Jesse Yu, James I. Griffin, Xiaolin Xu, Rodney J. Y. Ho

**Affiliations:** 1 Department of Pharmaceutics, University of Washington, Seattle, WA 98195, USA; linxiz@uw.edu (L.Z.); jesseyu73@gmail.com (J.Y.); griffj8@uw.edu (J.I.G.); alinxu@uw.edu (X.X.); 2 Department of Bioengineering, University of Washington, Seattle, WA 98195, USA

**Keywords:** metastatic breast cancer, nanoparticles, ICAM-1, targeting, combination therapy

## Abstract

Despite the availability of molecularly targeted treatments such as antibodies and small molecules for human epidermal growth factor receptor 2 (HER2), hormone receptor (HR), and programmed death-ligand 1 (PD-L1), limited treatment options are available for advanced metastatic breast cancer (MBC), which constitutes ~90% mortality. Many of these monotherapies often lead to drug resistance. Novel MBC-targeted drug-combination therapeutic approaches that may reduce resistance are urgently needed. We investigated intercellular adhesion molecule-1 (ICAM-1), which is abundant in MBC, as a potential target to co-localize two current drug combinations, gemcitabine (G) and paclitaxel (T), assembled in a novel drug-combination nanoparticle (GT DcNP) form. With an ICAM-1-binding peptide (referred to as LFA1-P) coated on GT DcNPs, we evaluated the role of the LFA1-P density in breast cancer cell localization in vitro and in vivo. We found that 1–2% LFA1-P peptide incorporated on GT DcNPs provided optimal cancer cell binding in vitro with ~4× enhancement compared to non-peptide GT DcNPs. The in vivo probing of GT DcNPs labeled with a near-infrared marker, indocyanine green, in mice by bio-imaging and G and T analyses indicated LFA1-P enhanced drug and GT DcNP localization in breast cancer cells. The target/healthy tissue (lung/gastrointestinal (GI)) ratio of particles increased by ~60× compared to the non-ligand control. Collectively, these data indicated that LFA1 on GT DcNPs may provide ICAM-1-targeted G and T drug combination delivery to advancing MBC cells found in lung tissues. As ICAM-1 is generally expressed even in breast cancers that are triple-negative phenotypes, which are unresponsive to inhibitors of nuclear receptors or HER2/estrogen receptor (ER) agents, ICAM-1-targeted LFA1-P-coated GT DcNPs should be considered for clinical development to improve therapeutic outcomes of MBCs.

## 1. Introduction

Breast cancer is the most common cancer in women worldwide and the second most common cancer overall. [1] Even with the best current therapies, breast cancer can recur and eventually spread to highly perfused lung tissues as metastatic breast cancer (MBC). Between 20% and 30% of patients with early breast cancers progress to MBC [2], the second leading cause of death among women in the USA. Among all MBCs, lung involvement contributes to 60–70% MBC mortality [3]. Therapy for MBC involves a multimodal strategy including surgery, radiotherapy and chemotherapy. Unlike the surgical resection of primary tumor mass early in diagnosis, the surgical removal of MBC tumor mass has been seen as a palliative. It is not clear whether surgical intervention for MBC can improve the survival and prognosis of such patients [4]. Systemic chemotherapy has been the core treatment strategy and crucial component of MBC treatments, but off-target toxicity to healthy tissues limits its potential impact [5]. Although combination therapy with multiple targets could provide a synergistic effect with enhanced efficacy and potentially reduced toxicity, the co-delivery of drugs and their synergies are limited by their different physicochemical and unsynchronous pharmacokinetic profiles, respectively.

Scientists have been investigating nanoparticles (NPs) to carry multiple drugs in the same particles for MBC. However, the ability to carry drugs with disparate physical qualities suitable for pharmaceutical preparation (such as hydrophobic and hydrophilic qualities) is limited. To be effective, the drug-combination nanoparticles (DcNPs) must also be selective for advancing MBC cells in vivo. To address these challenges, our laboratory has recently developed a targeted and long-acting DcNP composition that enables the incorporation of both hydrophilic and hydrophobic drugs at a scale suitable for pharmaceutical preparation. With this validated technology and process in place, we were able to incorporate both water-soluble gemcitabine (G) and water-insoluble paclitaxel (T) together in a novel DcNP platform, initially with multiple human immunodeficiency virus (HIV) drug combinations [6,7]. Subsequently, the DcNP technology is shown to enable the incorporation of G (soluble) and T (insoluble), together with two lipid excipients, to form a single nanosuspension (~60 nm in diameter) [8,9]. The GT in DcNPs, referred to as GT DcNPs, enhance the exposure of G and T in mice with the apparent half-life of G increased by 2.9-fold and the overall plasma exposure by 61-fold (G). For T, the half-life increased by 3.8-fold (T) [8,9]. Due to differential localization in advancing MBC in the lungs, a single intravenous (IV) dose of GT DcNPs could nearly eliminate lung nodules with a five times lower dose than a Cremophor EL (CrEL) drug-combination control and had a five-fold higher tissue-to-plasma ratio of G in tumor-bearing lungs compared to in healthy lungs [9]. However, at high doses, we observed some weight loss in mice that might be attributed to gastrointestinal (GI) toxicity, which could be overcome with a cancer cell-specific marker to localize and enhance the MBC exposure of GT DcNPs [8].

In searching for potential molecular targets, we found that intercellular adhesion molecule-1 (ICAM-1), an immunoglobulin superfamily glycoprotein located on the cell surface, is highly expressed in certain types of breast cancer cells (i.e., triple-negative subtypes) [10]. ICAM-1 plays an important role in tumor metastasis, spread, and disease progression [11,12]. Additionally, the expression of ICAM-1 has been observed in various types of cancers and is associated with advanced cancer stages [12,13,14]. Lymphocyte function-associated antigen 1 (LFA-1), part of the endothelial cell matrices within the vasculature, has been reported as the ligand of ICAM-1 [15,16,17]. An LFA-1 peptide mimic, ITDGEATDSGC (referred to as LFA1-P peptide), derived from the I-domain of the αL-subunit of LFA-1 integrin has been reported to inhibit LFA-1 and ICAM-1 interaction by binding to ICAM-1 (also preventing ICAM-1-mediated receptor internalization) [15,16,17]. This LFA1-P peptide displays receptor-mediated endocytosis, indicating its possible use as a targeting moiety for intracellular drug delivery. Indeed, it has been shown that LFA1-P-conjugated polymeric PLGA NPs containing proteolipid protein (PLP139–151) antigens for multiple sclerosis can target ICAM-1 and further alter T cell responses [18].

In this study, we have developed and evaluated the ability of ICAM-1-targeted GT DcNPs to enhance the localization of DcNPs and the two drugs, G and T, in MBC that expresses ICAM-1. ICAM-1 was targeted with LAF1-P peptide coated on GT DcNPs. In a 4T1 MBC lung-nodule model, we found that LFA1-P incorporation into GT DcNPs exhibited enhanced particle and drug localization in tumor laden lung tissues.

## 2. Materials and Methods

### 2.1. Materials

G free base (>99%; 2′,2′-Difluorodeoxycytidine; CAS 95058-81-4) and T (>99.5%; CAS 33069-62-4) were purchased from LC Laboratories (Woburn, MA, USA). 1,2-distearoyl-sn-glycero-3-phosphocholine (DSPC) and 1,2-distearoyl-sn-glycero-3-phosphoethanolamine-N-[amino (polyethylene glycol)-2000] (ammonium salt; DSPE-PEG2000; GMP grade) were purchased from Corden Pharma (Liestal, Switzerland). Indocyanine green (ICG; C_43_H_47_N_2_NaO_6_S_2_; sodium 2-[7-[3,3-dimethyl-1-(4-sulfonatobutyl) benz[e]indolin-2-ylidene] hepta-1,3,5-trien-1-yl]-3,3-dimethyl-1-(4-sulfonatobutyl) benz) was purchased from TCL America. Anhydrous ethanol was purchased from Decon Pharmaceuticals (King of Prussia, PA, USA). All the other reagents used were of analytical grade or higher. Rabbit anti-mouse ICAM-1 polyclonal antibody (cat. no. 16174-1-AP) was purchased from Proteintech Group (Rosemont, IL, USA). The HA58 mAb (mouse anti-human ICAM-1) was purchased from BioLegend, San Diego, CA, USA. Goat anti-mouse IgG antibody (Alexa Fluor^®^ 555; cat. No. 405324) was purchased from BioLegend, San Diego, CA, USA. Goat anti-rabbit IgG H&L (Alexa Fluor^®^ 555; cat. no. ab150078) was purchased from Abcam (Boston, MA, USA). Frozen 4T1 cells, MDA-MB-231 cells, L929 cells, and SK-BR-3 cells were purchased from the American Type Culture Collection (ATCC, Manassas, VA, USA). LFA1-P peptide with N-terminal palmitoylation and C-terminal amidation was customized by GenScript, Inc. (Piscataway, NJ, USA).

### 2.2. Preparation of LFA1-P and ICG Co-Loaded NPs

Lipid NPs co-loaded with LFA1-P peptide and ICG were prepared by thin film hydration and sonication. Briefly, DSPC, DSPE-mPEG2000, and ICG were dissolved in a mixture of ethanol at 60 °C. LFA1-P peptide was first dissolved in a mixture of chloroform/ethanol/methanol/water (*v*/*v*/*v*/*v*: 20/15/3/4) at 0.5 mg/mL and then added into a lipid mixture. The solvent was removed by rotary evaporation to form a thin film, which was vacuum-desiccated at least overnight to remove the residual solvent. The thin film was rehydrated in 0.45% NaCl with a 20 mM NaHCO_3_ buffer at 60 °C for 2 h with shaking. The NPs diameters were reduced to ~60 nm with a total of 15 min of water bath sonication at ~45 °C (5 min on and 5 min off for 3 cycles; Avanti Polar Lipids, Inc., Alabaster, AL, USA). The particles sizes were determined by a NICOMP 380 ZLS (Particle Sizing Systems, Santa Barbara, CA, USA). The preparation was performed in the dark, and direct light exposure was avoided during handling.

### 2.3. Preparation and Characterization of GT DcNPs

G, T, DSPC, and DSPE were dissolved in ethanol at 60 °C to prepare GT DcNPs. For ICG-labeled GT DcNPs, ICG powder was added into the mixture; for the GT DcNP-LFA1-P preparation, LFA1-P was added as described above. The solvent was removed by rotary evaporation to form a thin film and was desiccated in a vacuum overnight to remove the residual solvent. The thin film was rehydrated with PBS at 60 °C for 2 h with shaking. The suspension was sonicated by a water bath sonicator (5 min on and 5 min off for 3 cycles). The particles sizes were determined with a NICOMP 380 ZLS. Drug association efficiency (AE%) was determined by dialyzing 100 μL of the GT DcNP suspension (6–8K MWCO) against a 1000× volume (100 mL, pH = 7.4) of bicarbonate buffered saline for 4 h at room temperature. Drugs were extracted by acetonitrile, and the resulting drug concentrations were measured by a Shimadzu HPLC-UV system (Kyoto, Japan), as we have previously described [8,9]. Drug concentrations were determined for both the pre- and post-dialysis of GT DcNP suspensions to determine the degree of drug association to GT DcNPs, as the dialysis membrane of 6–8K MWCO only permitted the removal of drugs not associated with GT DcNPs. Zeta potential was characterized using a Zetasizer Nano-ZS (Malvern Instruments, Worcestershire, UK). The analyses were performed at room temperature. The pH values of all NP solutions for zeta potential measurements were 7.4 (dilution in a 20 mM HEPES buffer).

### 2.4. Cell Culture

4T1 cells, MDA-MB-231 cells, L929 cells, and SK-BR-3 cells were purchased from the ATCC and grown in Dulbecco’s Modified Eagle’s Medium (DMEM) or Roswell Park Memorial Institute Medium (RPMI) supplemented with 10% fetal bovine serum and 1% antibiotics. The cells were cultured in an incubator maintained at 37 °C and 5% CO_2_ with 95% humidity.

### 2.5. Immunofluorescence Analysis of the ICAM-1 Expression on the Cell Surface

The ICAM-1 expression levels in 4T1, MDA-MB-231, SK-BR-3, and L929 cells were detected by immunofluorescence using an ICAM-1 specific antibody. Cells (30,000/well) were seeded into a sterilized 8-well chamber slide. After overnight incubation, the cells were fixed for 10 min in 4% (*w*/*v*) paraformaldehyde/PBS at room temperature. A blocking solution (5% BSA in PBS) was added and incubated for 1 h at room temperature. After washing by PBS (3 times, 3 min/washing), an anti-ICAM-1 antibody (diluted in 1% BSA in PBS) was added to the wells. After 1.5 h of room-temperature incubation, the primary antibody was removed, and an Alexa 555-conjμgated secondary antibody (against rabbit or mouse primary antibodies, diluted in 1% BSA in PBS) was then added and incubated for 1 h at room temperature. The cells were then washed by PBS (3 times, 3 min/washing), the chamber wall was removed, and a mounting medium containing DAPI was added onto the slide. The ICAM-1 expression on cells was detected with an epifluorescence microscope (Zeiss, Oberkochen, Germany).

### 2.6. Cell-Binding Assay

4T1 cells were seeded on a 12-well plate at a density of 300,000/well. After the overnight incubation, particles were added into the wells at concentrations of 0.5 mM (lipid concentration), and the plates were incubated at 4 °C (a low temperature to prevent internalization). The cells were washed 3 times with 500 μL of cold PBS (4 °C). After media removal in wells, the cells were then dissolved into 100 μL of the solvent (100% DMSO). The resulting mixture was transferred into a 96-well microplate (black wall and clear bottom) for near-IR fluorescence measurement. The fluorescence measurements were performed on a Victor3 V 1420-040 Multilabel Plate Reader (Perkin Elmer; Waltham, MA, USA) with a tungsten-halogen continuous wave lamp (75 W; spectral range: 320–800 nm) and excitation (769 ± 41 nm) and emission (832 ± 37 nm) filters (Semrock; Rochester, NY, USA).

### 2.7. Preparation of GT CrEL Suspension

To prepare an equivalent GT drug combination for use as a control formulation, T was first dissolved in ethanol (20 mg/mL) and diluted by CrEL (*v*:*v*, 1:1; Sigma-Aldrich, St. Louis, MO, USA). The resultant T solution was further diluted 10-fold with PBS containing pre-dissolved G (hydrochloride salt, 12.65 mg/mL). The final concentrations of drug combination in the suspension were 10 mg/mL G and 1 mg/mL T. This control drug combination in the suspension was used in animal studies within the same day of preparation due to the instability of the drug combination in the suspension (precipitation of T in the suspension over time due to the high hydrophobicity; G had good solubility in the suspension).

### 2.8. Cytotoxicity Assay

4T1 cells were seeded into the wells of a 96-well plate (500 cells/well) at standard culture conditions of 5% CO_2_ in air at 37 °C. After overnight incubation, GT DcNP- LFA1-P, GT DcNPs, and free drug (GT-free drug combination) were added into the culture media at final concentrations of 0.128, 0.064, 0.032, 0.016, 0.008, 0.004, 0.002, 0.001, 0.0005, and 0.00005 μg/mL (each concentration was triplicated, and the experiments were performed three times independently.). The plate was returned to the incubator. After another 3 days of incubation, Alamar Blue was added directly into the culture media at a final concentration of 10%, and fluorescence intensity was measured after 1.5 h incubation according to the manufacturer’s manual.

### 2.9. In Vivo GT DcNP Targeting and Tumor Inhibition Study

Animal studies were conducted in accordance with University of Washington Institute of Animal Care and Use Committee (IACUC) approved protocols as well as with federal guidelines. Five-week-old female BALB/c mice were purchased from the Jackson Laboratory (Bar Harbor, ME, USA) and housed in an animal research facility for at least one week before use.

Six-week-old, female BALB/c mice were used in the targeting study. 4T1 cells transfected with luciferase and green fluorescence protein (GFP) (4T1-luc) were used for bioluminescence detections [8]. 4T1-luc (2 × 10^5^ cells) was suspended in 100 µL ice-cold HBSS and intravenously inoculated through mouse tail veins. The mice were inoculated with 4T1 cells and given a single IV dose of either a GT DcNP, GT DcNP-LFA1-P (1%), or GT DcNP-LFA1-P (2%) formulation on day 0 (n = 3). Mice and tissue imaging was conducted on day 10. The mice received 150 mg/kg of D-luciferin through intraperitoneal injections 10–15 min before imaging. Bioluminescence imaging parameters for living mice and tissue were set as follows: field of view, 12; excitation filter, closed; emission filter, open; exposure time, 120 s (living mice) and 60 s (lung tissue); binning factor, 4; f/stop, 2. Total 4T1-luc bioluminescence emissions from the living mice were integrated using Live Image software (PerkinElmer, Waltham, MA, USA).

In tumor inhibition effect studies, six-week-old, female BALB/c mice were inoculated with 2 × 10^5^ 4T1-luc cells IV in 100 µL HBSS on day 0. Three hours later, mice were given a single administration of saline, a CrEL-based-free drug combination, GT DcNPs, or GT DcNP-LFA1-P through IV injections (n = 5). The GT doses were 5/0.5 mg/kg G/T for CrEL and DcNP formulations. On day 14, the mice were imaged by in vivo imaging system (IVIS) and euthanized, and lungs were collected. Mouse lung tissue was fixed in 10% formalin and stored in 70% EtOH.

### 2.10. Drug Extraction from Plasma and Tissues

A liquid–liquid extraction was used to extract G and T from plasma or tissue homogenates according to our previous description [9]. Fifty microliters of sample was transferred into 1.5 mL tubes with or without dilution via a blank matrix to an appropriate concentration range. The samples were spiked with internal standards followed by the addition of 9 volumes of acetonitrile. The samples were then vortexed for 6 min and centrifuged at 4 °C for 15 min at 14,000 rpm. The supernatant was then removed and dried under nitrogen at 40 °C. The dried samples were reconstituted in 50 mL of the mixture with 20% methanol and 80% water.

### 2.11. Quantification of Drugs by LC-MS/MS

The drugs were quantified by a Shimadzu HPLC system coupled to a 3200 QTRAP mass spectrometer (Applied Biosystems, Grand Island, NY, USA) [9]. The HPLC system consisted of two Shimadzu LC-20A pumps, a DGU-20A5 degasser, and a Shimadzu SIL-20AC HT autosampler (Shimadzu Corporation, Kyoto, Japan). The mass spectrometer was equipped with an electrospray ionization (ESI) TurboIonSpray source. The system was operated with Analyst software, version 1.5.2 (ABSciex, Framingham, MA, USA). The chromatographic separation of G and T was achieved using a Synergi column (100 × 2.0 mm; 4 μm in particle size) (Phenomenex, Torrance, CA, USA) with an inline C8 guard column (4.0 × 2.0 mm) also from Phenomenex. The flow rate was set to 0.5 mL/min with a 5 mL sample injection volume. The mobile phase for separation consisted of pump A (20 mM ammonium acetate in water) and B (reagent alcohol). The gradient program used was as follows: pump B was maintained at 20% for 1.0 min, then increased to 97% at 2.0 min, held at 97% until 3.0 min, ramped to 3% by 4.0 min and held until 5.5 min. The needle was washed with isopropanol after each injection. Analytes were monitored using multiple-reaction monitoring (MRM) for positive ions. The following ion transitions were monitored, i.e., G (*m/z* 264.066→112.000) and T (*m/z* 854.266→286.200); a stable labeled isotope (C_8_^13^CH_12_ClF_2_N^15^N_2_O_4_; *m/z* 267.067→115.100) was used as an internal standard for G; docetaxel (*m/z* 830.312→549.3) was used as an internal standard for T.

### 2.12. Statistical Analysis

Targeting and inhibition data were presented as the arithmetic mean ± SD. The number of the mice in all groups ranged from 3 to 5. Statistical analysis was performed using GraphPad Prism 7.04 (GraphPad Software Inc., San Diego, CA, USA). Statistical comparisons were performed using students’ *t*-tests, and one-way ANOVA was used to determine statistical significance for multiple treatment groups across studies. A *p*-value of ≤0.05 was considered statistically significant.

## 3. Results

### 3.1. Evaluation of ICAM-1 Expression on Human and Mouse Breast Cancer Cells

**Figure 1:**
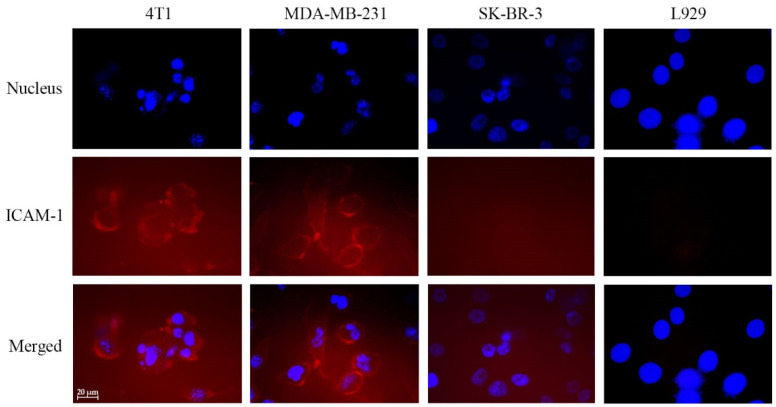
Immunofluorescent imaging of intercellular adhesion molecule-1 (ICAM-1) expression in 4T1, MDA-MB-231, SK-BR-3, and L929 cells. The cells were seeded into chamber slides (30,000 cells/well) and incubated overnight. The cells were fixed with paraformaldehyde, quenched and stained for ICAM-1 detection (red). The cells nuclei were stained with DAPI (blue). The scale bar in the figure is 20 mm. Each picture was chosen as representative from a triplicated experiment.

To investigate whether breast cancer cells express ICAM-1, which would be used for targeting drug combinations in GT DcNPs, we evaluated a LFA1-P peptide mimic of ICAM-1 ligand: LFA-1 peptide immobilized on GT DcNPs. To do so, we first used a fluorescently labeled the ICAM-1 antibody to verify the antigen expression on a few selected breast cancer cell lines and normal cells. As presented in Figure 1, we found that mouse 4T1 breast cancer cells expressed ICAM-1 while mouse fibroblast L929 cells did not. Among the four tested, the mouse 4T1 and human MDA-MB-231 breast cancer cells exhibited significant cell surface ICAM-1 expression as evident in a high degree of antibody fluorescence. Conversely, another two breast cancer cell SK-BR-3 and fibroblast L929 did not express significant levels of ICAM-1 (the quantified fluorescence signals in images were plotted in Supplementary Information, Figure S1). These results indicated that 4T1 and human MDA-MB-231 breast cancer cells expressed ICAM-1 and that this antigen may be used for cancer cell targeting studies for DcNPs containing G and T (referred to as GT DcNPs). More human breast cancer cell lines have been previously investigated for ICAM-1 expression (e.g., MCF-7 which is HR^+^/HER2^−^ has low ICAM-1 expression among other cell lines tested), and it was found that the cancer invasiveness was positively correlated with the ICAM-1 expression level [19].

### 3.2. Effect of Increasing the LFA1-P Peptide Density on NPs to Enhance the Binding to ICAM-1-Expressed 4T1 Breast Cacner Cells

To evaluate the ability of ICAM-1-binding peptide LFA1-P coated on NPs to bind cancer cells overexpressing ICAM-1, we incorporated LFA1-P to GT DcNPs, a previously described formulation [8,9]. GT DcNP was characterized as a non-liposome lipid nanoparticle without bilayer membrane structure and with elongated discoid shape as examined by transmission electron microscopy (length ~50 nm, width ~15 nm, [9]). The density of LFA1-P was first optimized in drug-free NPs in order to find the best ratio for the strongest targetability. The LFA1-P peptide was modified with N-terminal palmitoylation and C-terminal amidation to allow the incorporation of the peptide to the GT DcNP hydrophobic particle core. Amidation restored the charge status, and the palmitoyl group allowed embedding into hydrophobic regions of lipid chains within GT DcNPs. ICG, an infrared fluorescence dye, which is an FDA-approved agent for human use, was used as a marker for DcNPs. The molar ratio of lipid excipients—ICG-DSPC:DSPE2000:ICG molar ratio—was fixed at 90:10:1. We found that under this condition, ICG was 100% bound to NP (through a filtration test with a 10 kDa spin filter which no free ICG flowed through). The sizes of all particles with different LFA1-P contents were tested to have diameters of ~60 nm. Zeta potential values ranged from −10 to −16 mV. The size and zeta potential measurements indicated that these NPs with different LFA1-P densities (LFA1-P-to-total lipids molar ratio: 0~5%) had no significant difference in their physical size and charge properties.

**Figure 2:**
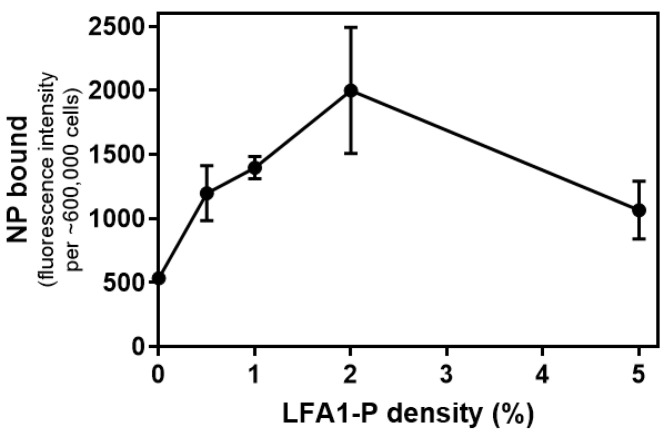
Effects of LFA1-P density on 4T1 cellular uptake of indocyanine green (ICG)-labeled NPs. 4T1 cells were seeded on a 12-well plate (300,000 cells/well) and incubated overnight. ICG-labeled NPs with 0.5%, 1%, 2%, and 5% LFA1-P of 0.5 mM (lipid concentration) were added into wells, and plates were incubated at 4 °C for 1 h. ICG fluorescence intensity was measured in a micro-plate reader, and the values are presented as mean ± SD of the replicates.

We then used ICG fluorescence to investigate quantitatively the role of the LFA1-P mole percentage in NPs on the binding ability of the NPs to 4T1 cells. As shown in Figure 2, the ICG fluorescent signal increased with the amount of NPs bound to the cell surface during 1 h incubation at 4 °C. Since endocytosis is inhibited at 4 °C [20,21], the NP amount on the cell surface can represent the binding ability of the NPs. Figure 2 shows the relationship between the density of LFA1-P in NPs and the intensity of the fluorescence signal. It can be seen that NPs with LFA1-P had better targetability than NPs without LFA1-P. In addition, when LFA1-P density was less than 2%, the LFA1-P density and the NP-binding capacity were positively correlated. However, when the density of LFA1-P continued to increase, the binding capacity of NPs began to decrease. When the LFA1-P density was increased to 5%, the binding capacity of NPs was similar to that at the 1% LFA1-P density. The in vitro NP-binding ability to a fixed number of 4T1 cells was strongest with an LFA1-P density of 2%. Therefore, we selected LFA1-P densities of both 1% and 2% for further in vitro and in vivo studies. To further confirm the binding specificity, we also tested the binding ability of NPs to 4T1 cells with/without the ICAM-1 antibody pre-incubation. The results showed that the blocking of ICAM-1 by the antibody decreased the signal of the NPs binding to 4T1 cells by 60.0%, further confirming that the NP–cell binding was ICAM-1-dependent (Supplementary Information, Figure S2).

### 3.3. Evaluation of Cellular Potency of GT DcNP, GT DcNP-LFA1-P, and GT-Free Drug Combination in 4T1 Cells

We next investigated the effects of LFA1-P on GT DcNPs ability to inhibit ICAM-1-expressed breast cancer cell growth. GT DcNPs were prepared as a previously reported process [8,9], and LFA1-P was added during the assembly of G and T together with two lipid excipients. We firstly tested the potential synergistic effect of the GT combination on MDA-MB-231 cells and the obtained the combination index of 0.9 (slightly synergistic; Supplementary Information, Table S1) using the Chou–Talalay formula [22]. The synergistic effect of the G and T combination was also reported elsewhere both in in vitro studies and in clinical trials [23,24]. The molar ratio of lipid excipients and drugs—DSPC:DSPE_2000_:G:T:ICG molar ratio—was 90:10:81:2.5:1 according to the previous studies [8,9], and the molar ratio of LFA1-P to lipid was 100:1 or 100:2. In this study, ICG was used as a DcNP marker, and IC was verified to be 100% associated. Both sizes of GT DcNPs and GT DcNP-LFA1-P were ~60 nm, the in vitro apparent DcNP% associations were ~12% and ~100% for G and T, respectively (~88% release of G and ~0% for T within 4 h under sink conditions). Despite of low in vitro association for G, the GT DcNP showed high drug association in vivo [9]. The results indicated that LFA1-P could be incorporated with GT DcNPs and that the products were stable and had no significant difference from GT DcNPs. All DcNPs were physicochemically stable (size, drug association, etc.) over at least 1 month at room temperature (6 months for ICG-free GT DcNP). Furthermore, the particle sizes of all NPs prepared in multiple batches were shown to be reproducible and meet the requirements of IV-injectable solutions.

**Figure 3:**
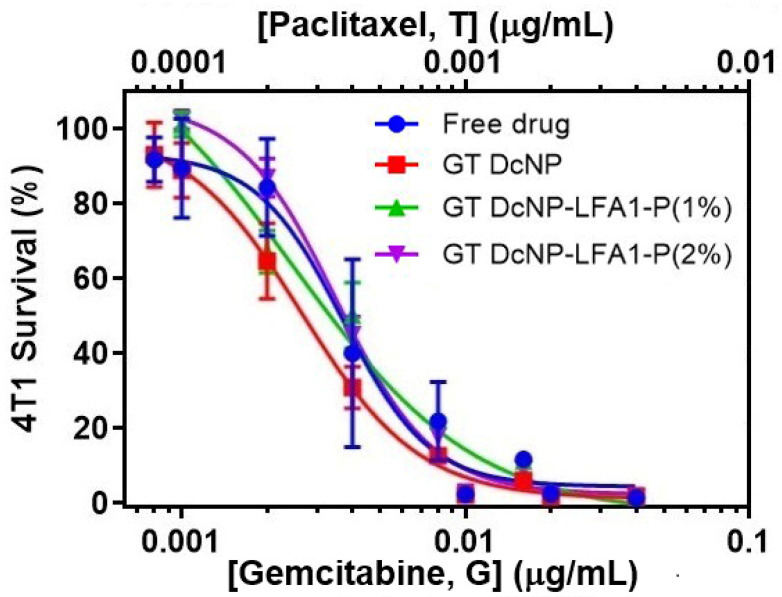
Dose–response curves of different drug combination dosages on 4T1 cell viability. Cell viability was evaluated by an Alamar Blue metabolic cell viability assay after 3 days of incubation with the GT DcNP, GT DcNP-LFA1-P, or free drugs as a GT combination. The G and T (fixed *w*/*w* ratio at 10:1) concentrations in the combination are indicated by the bottom and top x-axes, respectively.

With a selected GT DcNP-LFA1-P formulation, we investigated the efficacies of the GT DcNP-LFA1-P and the G T DcNP. The cellular potency of the GT DcNP, the GT DcNP-LFA1-P (1%), the GT DcNP-LFA1-P (2%), and the GT-free drug combination were tested in 4T1 breast cancer cells, expressing ICAM-1. Table 1 summarizes the half-maximal inhibitory concentration (IC_50_) values of all the different formulations. The IC_50_ or half-maximal inhibitory concentration is a measure of drug potency against specific cells. The concentration was based on the concentration of G in the NPs in each experiment, and the concentration of T was 1/10 of G. Figure 3 shows the IC_50_ of each drug for 4T1 breast cancer cells. We found that the GT DcNP-LFA1-P (1%) had the lowest IC_50_ value of 2.87 ng/mL, the GT DcNP have a slightly higher IC_50_ value recorded at 3.86 ng/mL, and the GT DcNP-LFA1-P (2%) had the highest IC_50_, which was 5.45 ng/mL. The efficacy of the free drug combination was higher than the GT DcNP-LFA1-P (2%) but lower than the GT DcNP and the GT DcNP-LFA1-P (1%). However, the IC_50_ values of the GT DcNP, the GT DcNP-LFA1-P (1%), the GT DcNP-LFA1-P (2%), and the GT-free drugs were in a similar range and had no statistical difference. The results indicated that incorporating 1% ICAM-1-binding peptide LFA1-P may provide a trend toward lower IC_50_ values in inhibiting 4T1 breast cancer cells, while increasing the peptide to 2% may produce lower potency. However, under static cell incubation conditions, the incorporation of peptides did not produce significant differences among the tested compositions. The GT in DcNPs with or without LFA1-P appeared to be biologically active.

### 3.4. Enhancement of In Vivo Targeting by GT DcNP-LFA1-P with a 4T1 Lung Metastasis Model

**Figure 4:**
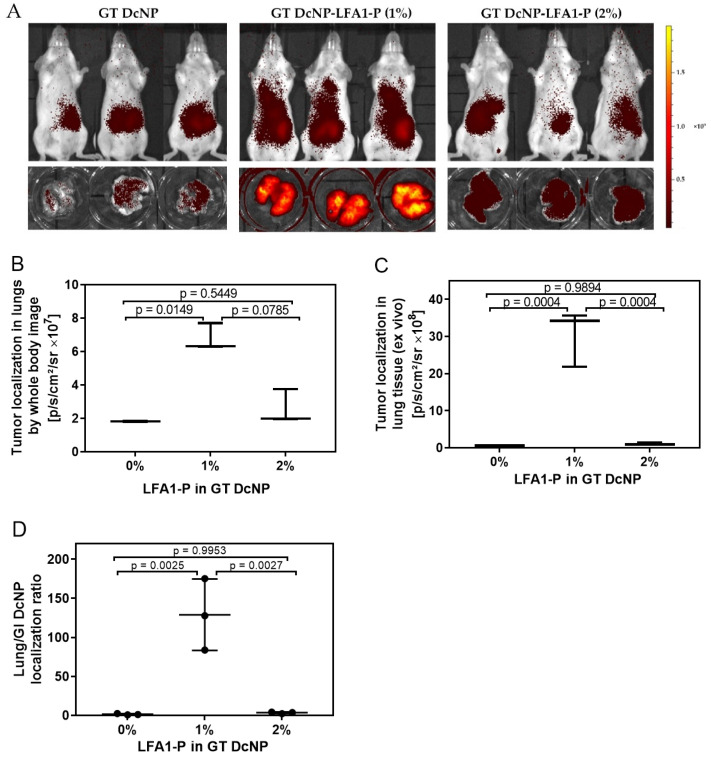
Effects of LFA1-P on the targeting of ICG-labeled GT DcNPs in 4T1 breast cancer lung metastasis models in mice. The mice inoculated with 4T1-luc breast cancer cells were administered with a single dose of ICG-labeled GT DcNP-LFA1-P (1%) and GT DcNP-LFA1-P (2%) (containing 50/5 mg/kg GT, IV). These mice were imagined for ICG fluorescence and presented as whole-body images (panel **A**), lung ICG intensity under whole-body image (panel **B**), and the ex vivo lung ICG intensity after removal of lung tissues (panel **C**). The ratio of the GT DcNP localization in GI and lungs was presented as the gastrointestinal (GI)/lung ICG intensity (panel **D**). The effects of LFA1-P density on the GT DcNP were determined with 0, 1%, and 2% of the total lipid excipient concentration of the ICG-GT DcNP, as described in each panel. The data are expressed as mean ± SD (n = 3). *p*-values were obtained from two-tailed *t*-tests.

**Figure 5:**
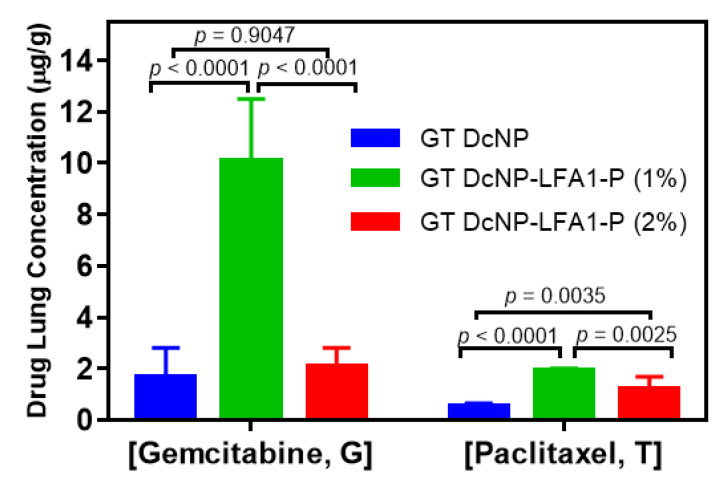
Effects of the LFA1-P content on the G and T lung accumulation of ICG-labeled GT DcNPs in 4T1 metastatic breast cancer mice. The mice inoculated with 4T1-luc breast cancer cells were administered with a single dose of ICG-labeled GT DcNP-LFA1-P (1%) or GT DcNP-LFA1-P (2%) (containing 50/5 mg/kg GT; IV). The G and T accumulation in lung tissues were quantified by LC-MS/MS. The data are displayed as mean ± SD (n = 3). *p*-values were derived with two-tailed *t*-tests.

To further validate LFA1-P-mediated targeting and drug delivery into ICAM-1-overexpressing cancer metastasis in vivo, we established a lung metastatic model of 4T1 with our previous method [8,9]. We first used 50/5 mg/kg (GT) based on the current clinical dose to compare the targetability of the GT DcNP-LFA1-P (1% and 2%) with the peptide-free GT DcNP (0%). Ten days after the mice were inoculated with 4T1 cells (tumor signal was sufficient to be detected by IVIS live imaging), a single IV dose of either the GT DcNP, the GT DcNP-LFA1-P (1%), or the GT DcNP-LFA1-P (2%) formulations was administered. Distinct from a single large tumor mass in typical subcutaneous models, the nodules were wide distributed, small in size and difficult to separate from healthy tissue for both drug and fluorescence signal analysis. We, thus, used the whole lung tissue for these analyses. The intensity of the GT DcNP fluorescence signal in mouse lungs was obtained by both live and ex vivo tissue imaging (Figure 4A). The quantification of lung ICG (DcNP marker) fluorescence intensities is shown in Figure 4B (live, chest area) and 4C (ex vivo, lungs). The results indicated that the lung ICG fluorescence intensity of the GT DcNP-LFA1-P (1%) was ~2.6 times (live imaging) and ~40 times (ex vivo imaging) higher than that of the GT DcNP-LFA1-P (2%). The drug (G and T) distributions in the lungs were analyzed by LC-MS/MS. The concentrations of G and T in lungs of 4T1 mice injected with GT DcNP-LFA1-P (1%) were determined to be 6.03- and 3.78-fold that of the ligand-free GT DcNP counterpart (Figure 5). In contrast, in the lung concentrations of G and T in mice treated with a higher LFA1-P density, the GT DcNP-LFA1-P (2%) was only 1.24 and 2.36 times higher than the control GT DcNP without LFA1-P targeted to ICAM-1. Furthermore, the GT accumulation ratio in metastatic lungs by GT DcNP-LFA1-P (1%) was close to 10:1, reflecting the original fixed dose formulation ratio and and high in vivo drug association with lipids, consistent with our previously reported finding [9]. Because GI side effects occur at high doses in humans treated with G [25,26] and in our previous dose–response study [8], it is a possible site of toxicity although we did not observe any GI abnormality in current experimental conditions. We then analyzed the selectivity of target vs. healthy tissues of the GT DcNP-LFA1-P by calculating the ex vivo bioluminescence signal ratios in metastatic lungs and GI. Impressively, the GT DcNP-LFA1-P (1%) increased the ratio by 57.45 compared to the no-ligand GT DcNP (Figure 4D). Collectively, these data suggested that a coating density at 1%, compared to at 2%, LFA1-P on the GT DcNP provided a higher degree of the GT DcNP localization in 4T1 breast cancer cells in the lungs, and both the DcNP and drug analyses verified LFA1P-mediated targeting effects.

### 3.5. Effect of LFA1-P on GT DcNPs to Enhance the Inhibitory Effect of 4T1 Metastatic Nodules in the Lungs

**Figure 6:**
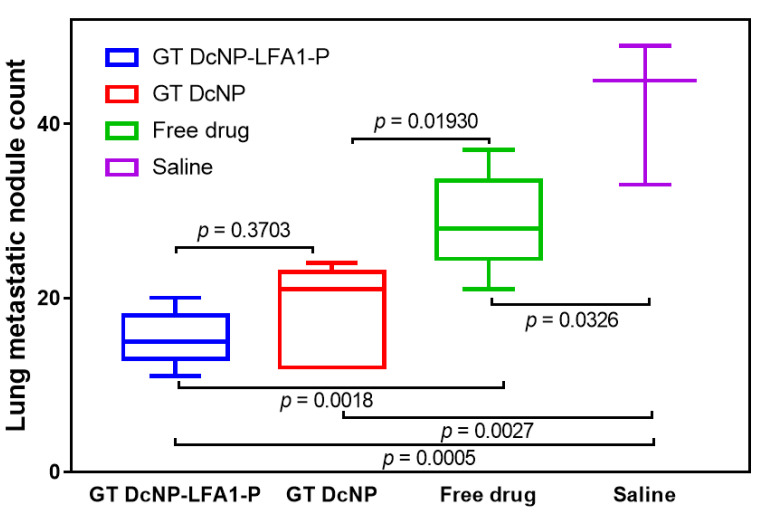
Effect of LFA1-P on GT DcNPs to enhance G and T combinations to inhibit 4T1 metastatic breast cancer nodules in the lungs. The mice inoculated with 4T1-luc via tail vein were administered with a 5/0.5 mg/kg GT in the GT DcNP, the GT DcNP-LFA1-P (1%), and the GT-free drug combination (CrEL) as a single IV dose (3 h post-inoculation, n = 5). On day 14, the total 4T1 metastatic tumor nodules in the lung tissues for each treatment and group were calculated as mean ± SD. *p*-values were obtained from two-tailed *t*-tests between groups.

To investigate whether the ICAM-1-targeted GT DcNP-LFA1-P can improve overall therapeutic outcomes of GT DcNPs, we evaluated the effect of GT DcNP-LFA1-P on inhibiting 4T1 lung metastasis. The mice inoculated with 4T1 cells were given a single IV dose of GT in the CrEL, the GT DcNP, or the GT DcNP-LFA1-P forms. A low GT dose (5/0.5 mg/kg) was chosen to discern the targeting effect of the GT DcNP-LFA1-P for GT DcNPs, as this dose was previously shown to partially inhibit 4T1 lung tumor formation when given in the GT DcNP dosage form [8,9]. We treated 4T1 mice at 3 h post-tumor-inoculation with an intent to discern the targeting of LFA1-P in GT DcNPs, compared to that without LFA1-P (i.e., GT DcNPs alone) and evaluated the formation of lung metastasis nodules at day 14. The 4T1 breast cancer expressed luciferase, which allowed the tracking of tumors based on the luciferase bioluminescence signal intensity. As the model was to mimic the condition of the remaining metastatic cells advancing through blood to distinct sites (lungs) after surgery, the primary tissue (mammary gland) was not examined (because no cancer cell was implanted). As shown in Figure 6, a single dose of the GT DcNP or the GT DcNP-LFA1-P could significantly inhibit the establishment and growth of 4T1 metastatic cancer in the lungs more than free drug combinations (CrEL) or a saline control. The GT DcNP-LFA1-P exhibited a trend toward more tumor than the GT DcNP (by 11%). Due to the small sample size, the data variation did not reach statistical significance (*p* > 0.05). However, compared to free GT, the GT DcNP-LFA1-P exhibited highly significant treatment effects (*p* = 0.01; GT DcNP-LFA1-P vs. free drug), and a lower degree of significance for GT DcNPs (*p* = 0.04 GT DcNP vs. free drug). Furthermore, the ex vivo metastatic lung images taken by a dissection microscope also indicated less and smaller nodules by the GT DcNP-LFA1-P group than the control group (Supplementary Information, Figure S3).

Collectively, these data suggested that a single low dose of the GT DcNP-LFA1-P may provide a higher potency and reduce GI untoward effects compared to a relatively potent GT DcNP counterpart. Both the GT DcNP and GT DcNP-LFA1-P were more effective than free drugs formulated in CrEL emulsions. These encouraging data collected from the small number of animals in this study may need to be followed with a dose–response study, powered with a larger sample size.

It was noteworthy that we did not observe any ICAM-1-related adverse effects in this study, such as toxic effects on healthy cells expressing ICAM-1 or effects related to leukocyte–endothelial cell interaction. This was likely due to the high abundance of ICAM-1 on cancerous tissues compared to on healthy tissue, low doses applied, or short observation time frame in this study. Although reported studies in the literature as well as our own observations all showed positive impact on cancer-specific drug delivery mediated by ICAM-1 [27,28,29], the ICAM-1-related adverse effects need to be further studied with different time frames, various doses, and function-specific assays.

## 4. Discussions

With the use of LFA1-P peptide to mimic the ICAM-1-targeted endogenous ligand LFA-1 and the demonstrated expression of breast cancer cell 4T1, we have successfully developed and characterized ICAM-1-targeted DcNPs containing G and T. We found that by coating DcNPs containing GT with 1% LFA1-P, the GT DcNP LFA1-P can be made reproducibly in nano-drug particles with an approximately 60 nm diameter. G and T in DcNPs were biologically active both in vitro and in vivo. The incorporation of LFA1-P has enhanced particle accumulation in the 4T1 metastatic lungs of mice as determined both by the particle marker and GT drug analysis. In addition, the lung tissues of mice with G and T inoculated with 4T1 breast cancer cells exhibited a similar G/T drug ratio to that of the original dosage form, suggesting that the two drugs were delivered to target cells and tissues as a collective GT DcNP unit. The drug analysis of the tumor in GI tissue indicated that the incorporation of LFA1-P has reduced drug localization in the GI while enhancing tumor drug accumulation. Thus, LFA-1P may provide a higher therapeutic index of G and T in drug combination, which is currently used to treat patients with advanced MBC.

For breast cancer treatment, estrogen receptor (ER, expressed in ~70% of all breast cancer) and HER2 (expressed in ~15% of all breast cancer) are the key drug targets for most available drugs [30]. For example, FDA-approved drugs including trastuzumab, pertuzumab, lapatinib, and neratinib target HER2, and thus they are used to treat HER2-positive breast cancer patients [31,32,33,34,35]. The mammalian target of rapamycin (mTOR) inhibitor—everolimus—and the CDK4/6 inhibitors—palbociclib and ribociclib—have been approved to treat breast cancer patients who are HER2-negative and hormone receptor (HR)-positive [36,37,38,39,40]. Triple-negative breast cancer (TNBC), which lacks the expression of both HR and HER2 [11,41], has chemotherapy as the main treatment approach with new approvals of targeted therapy and immune checkpoint inhibitors added [39]. Since the proliferation of cancer cells is related to angiogenesis and tyrosine kinase, anti- vascular endothelial growth factor (VEGF) antibodies such as bevacizumab, anti-vascular endothelial growth factor receptors (VEGFR) antibodies such as ramucirumab, and tyrosine kinase inhibitors such as sunitinib are also approved for TNBC treatment [42,43,44,45,46,47]. However, none of these shows substantial survival benefits, and drug resistance is still a big problem for targeted cancer therapy [39,40]. Under these circumstances—when patients are at advance breast cancer with metastatic presentation, chemotherapeutic combinations such as G and T are prescribed. However, dose-limiting toxicity typically prevents patients from completing the course of therapy or reduces the dose and thus limits the therapeutic potential of the G and T drug combination intended to maximally inhibit tumor growth and eliminate MBC in patients.

With DcNPs composed of two lipid excipients able to stabilize unlikely water-soluble G and water-insoluble T together in one nanoparticulate form, we were able to enhance the accumulation of GT in metastatic 4T1 breast cancer cells in a mouse MBC model. The DcNP approach has provided long-acting and sustained GT drug levels in mice, which may also be related to an improved therapeutic index of the GT drug combination [8,9]. In a previous study, we found that major untoward effects of GT DcNPs, as with a high dose of free G, are GI side effects related to the overall weight loss. With the addition of ICAM-1 ligand LFA1-P, we were able to direct GT DcNPs to 4T1 MBC cells in lung tissues to further increase drug accumulation in cancer cells and reduce the toxicity caused by off-target effects [8,9].

It is worth noting that the overexpression of ICAM-1 has been reported in TNBC [14,27]. As TNBC lacks the expression of the ER, the progesterone receptor (PR), and HER-2 [48], regular targeted therapy cannot apply to TNBC treatment. This is why the commonly used methods for treating TNBC are mainly surgery, chemotherapy, and radiotherapy [41,49]. Our novel GT DcNP-LFA1-P can actively target the ICAM-1 receptor on the surface of TNBC and thereby provide a targeted TNBC treatment, which has been also demonstrated in previous research [12,14].

Additional studies may be needed to explore the role of metastatic tumor establishment, providing a window of opportunity for targeted GT DcNPs to identify the time and dosage optimal for applying targeted vs. untargeted GT DcNPs for clinical translation. In addition, dose–response studies may be needed to define the full range of efficacy and safety and to define the therapeutic index. The targeted GT DcNPs are not limited to a 4T1 cell model. It could be used in any cell that highly expresses ICAM-1, such as human MDA-MB-231 cells [11]. This targeted GT DcNP strategy may be useful to provide a higher safety and therapeutic response for other highly potent drugs in use or those in development for treating incurable MBCs. It could also be used to develop longer lasting and more effective drug-combination therapies for other diseases where drug combinations are needed to find a cure or effective treatment including chronic and acute lung infections.

## 5. Conclusions

In conclusion, we have developed a strategy to target NPs containing both G and T to the MBC expressing the ICAM-1 receptor. To target ICAM-1, a peptide fragment of ICAM-1 ligand LFA1 (LFA1-P) was incorporated into GT DcNPs. We prepared a stable formulation GT DcNP-LFA1-P and evaluated its targeting characteristics in cells and in a MBC model in mice. LFA1-P could enhance the GT DcNP binding with ICAM-1-positive 4T1 cells in an in vitro binding assay. The cellular potency in 4T1 cells appeared to be similar between targeted and non-targeted GT DcNPs and GT-free drugs, indicating the retention of drug functions. In a 4T1 lung metastatic mouse model, we observed that adding LFA1-P to GT DcNPs significantly increased both G (4.9-fold) and T (1.6-fold) accumulations in metastatic lung tissues. A preliminary treatment study in the 4T1 model with a low dose indicated that ICAM-1-targeted GT DcNP-LFA1-P may provide additional metastatic tumor nodule reduction beyond that of non-targeted GT DcNPs and GT-free drugs. The incorporation of LFA1-P also enhanced the metastatic tissue-to-gut tissue drug ratio, which may lead to a reduction in GI untoward effects. These promising results warrant further development of GT DcNPs targeting ICAM-1-positive breast cancer (and maybe other cancers) to improve clinical outcomes with higher margin of safety.

## Figures and Tables

**Table 1:** Half-maximal inhibitory concentration (IC_50_) values of the gemcitabine and paclitaxel drug-combination nanoparticle (GT DcNP), GT DcNP-LFA1-P (1%), GT DcNP-LFA1-P (2%), and GT-free drug combination on 4T1 cell viability tested from a 3-day static incubation.

	Half-Maximal Inhibitory Concentration (IC_50_ in ng/mL, Mean ± SD)			
	**GT DcNP**	**GT DcNP-LFA1-P (1%)**	**GT DcNP-LFA1-P (2%)**	**GT-Free Drug Combination**
G and T-combined dose (*w*/*w*, 10:1)	3.86 ± 2.40	2.87 ± 0.35	5.45 ± 3.07	5.03 ± 2.48

## Data Availability

The data presented in this study are within the article and supplementary material, or on request from the corresponding author.

## Supplementary Materials

The following are available online at https://www.mdpi.com/article/10.3390/pharmaceutics14010089/s1, Figure S1: Mean intensity values of ICAM-1 fluorescence signal in Figure 1 for different cells. The values were quantified by Image J, Figure S2: Effects of ICAM-1 blocking on 4T1 cellular binding of ICG-labeled nanoparticles. 4T1 cells were seeded on a 12-well plate (300,000 cells/well) and incubated overnight. Blocking group was pre-incubated with ICAM-1 polyclonal antibody at 37 °C for 30 min (11 μg/mL). After removal of antibody, ICG-labeled NP with 1% LFA1-P of 1 mM (lipid concentration) were added into wells, and plates were incubated at 4 °C for 1 h. Cells were then lysed by DMSO and the ICG Fluorescence intensity was measured in a micro-plate reader and the values are presented as mean ± SD of the replicates, Figure S3: Representative lung image with different treatments in 4T1 tumor inhibition study in correlation with Figure 6. After the mice were euthanized at day 14, tissues were fixed with 10% formalin for at least 24 h, and then stored in 70% ethanol. The images were taken by a dissection microscope. Dark blue dashed circles in the figure indicate nodule margins in the lungs, Table S1: IC_50_ and combination index (CI) calculation of GT combination in MDA-MB-231 cells after a 5-day incubation. CI was calculated based on the Chou-Talalay formula (Cancer Res., 2010, 70(2), 440–446).
