# Supplementary material for: ICAM-1 Targeted Drug Combination Nanoparticles Enhanced Gemcitabine-Paclitaxel Exposure and Breast Cancer Suppression in Mouse Models
Source: Pharmaceutics. 2021 Dec 31;14(1):89. doi: 10.3390/pharmaceutics14010089 (PMC8779833; doi:10.3390/pharmaceutics14010089)
Supplement: Supplementary file 1 [file pharmaceutics-14-00089-s001.zip › pharmaceutics-1512085-supplementary.pdf]

pharmaceutics

MDPI

Supplementary Materials: ICAM-1 Targeted Drug Combination Nanoparticles Enhanced Gemcitabine-Paclitaxel Exposure and Breast Cancer Suppression in Mouse Models

Linxi Zhu, Qingxin Mu, Jesse Yu, James I. Griffin, Xiaolin Xu and Rodney J. Y. Ho

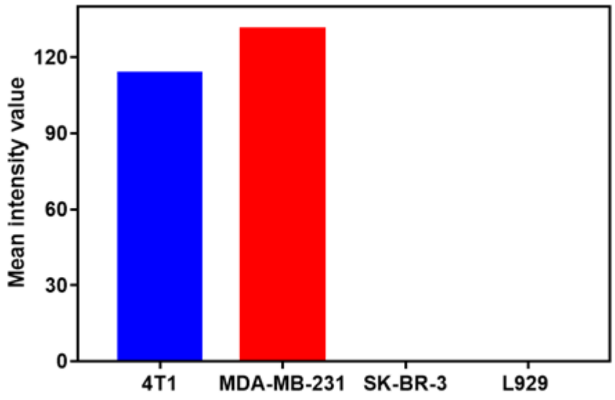

Figure S1. Mean intensity values of ICAM-1 fluorescence signal in Figure 1 for different cells. The values were quantified by Image J.

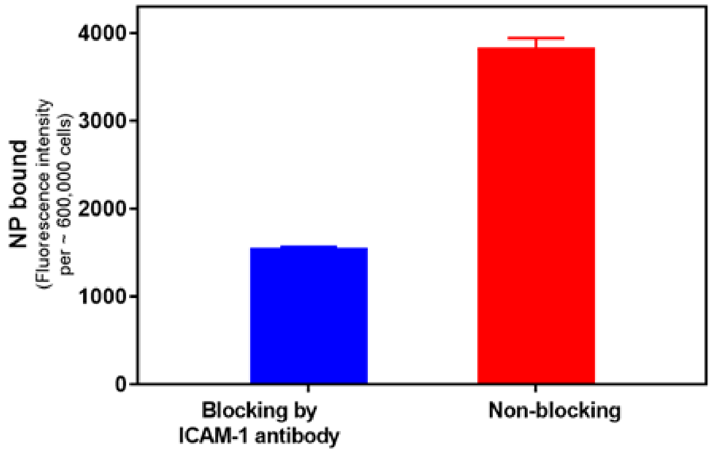

Figure S2. Effects of ICAM-1 blocking on 4T1 cellular binding of ICG-labeled nanoparticles. 4T1 cells were seeded on a 12-well plate (300,000 cells/well) and incubated overnight. Blocking group was pre-incubated with ICAM-1 polyclonal antibody at 37 °C for 30 min (11 μg/mL). After removal of antibody, ICG-labeled NP with 1% LFA1-P of 1 mM (lipid concentration) were added into wells, and plates were incubated at 4 °C for 1 h. Cells were then lysed by DMSO and the ICG Fluorescence intensity was measured in a micro-plate reader and the values are presented as mean ± SD of the replicates.

Table S1. IC_{50} and combination index (CI) calculation of GT combination in MDA-MB-231 cells after a 5-day incubation. CI was calculated based on the Chou-Talalay formula (Cancer Res., 2010, 70(2), 440-6.).

	IC_{50} (ng/mL, single drug)	IC_{50} (ng/mL, in combination, 10/1 w/w)
Gemcitabine	0.92	0.77
Paclitaxel	1.27	0.077
Combination index at IC_{50} (CI)	n/a	0.9

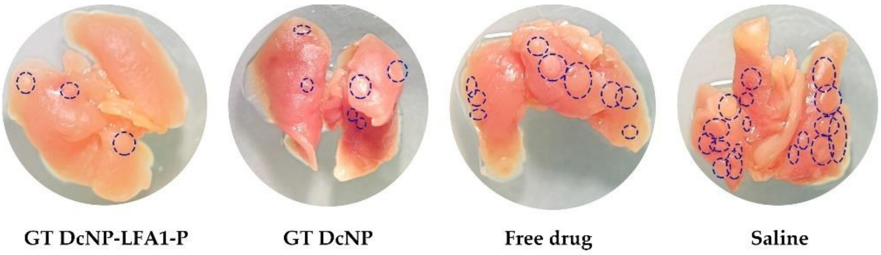

Figure S3. Representative lung image with different treatments in 4T1 tumor inhibition study in correlation with Figure 6. After the mice were euthanized at day 14, tissues were fixed with 10% formalin for at least 24 h, and then stored in 70% ethanol. The images were taken by a dissection microscope. Dark blue dashed circles in the figure indicate nodule margins in the lungs.
